# How do Smokers in a Snus-Prevalent Society Consider E-cigarettes, Snus, and Nicotine Replacement Therapy Products as Relevant Replacements for Cigarettes in the Event They Should Stop Smoking?

**DOI:** 10.1093/ntr/ntad113

**Published:** 2023-07-06

**Authors:** Tord Finne Vedøy, Karl Erik Lund

**Affiliations:** Department Alcohol, Tobacco, and Drugs, Norwegian Institute of Public Health, Oslo, Norway; Department Alcohol, Tobacco, and Drugs, Norwegian Institute of Public Health, Oslo, Norway

## Abstract

**Introduction:**

Around 50 percent of the tobacco in Norway is consumed in the form of snus, a smokeless oral tobacco. We examined Norwegian smokers’ openness, and thereby the potential reach, to use e-cigarettes, nicotine replacement therapy products (NRT), and snus in the event of quitting smoking, in a society where snus use is common.

**Methods:**

Using data from an online survey of 4073 smokers from 2019 to 2021, we calculated predicted probabilities of smokers’ being open, undecided, and not open to use e-cigarettes, snus, and NRT in the event they should quit smoking.

**Results:**

Among daily smokers, the probability of being open to use e-cigarettes in the event of quitting smoking was .32. The corresponding probabilities for using snus and NRT were .22 and .19. Snus was the product with the highest probability of *not* being open (.60). NRT had the highest probability of being undecided (.39). Among smokers who had never used e-cigarettes or snus, the probabilities of being open were .13 for e-cigarettes, .02 for snus and .11 for NRT.

**Conclusions:**

In a snus-friendly norm climate where smokers have traditionally used snus as an alternative to cigarettes, the probability of using e-cigarettes in the event of smoking cessation was higher compared to both snus and NRT. However, among smokers who had never used e-cigarettes or snus, the likelihood of being open to use of NRT was similar to e-cigarettes, and higher than snus, which suggests that NRT may still play a role in smoking cessation.

**Implications:**

In a snus-prevalent country in the endgame phase of the cigarette epidemic, where robust infrastructure for tobacco control in combination with the availability of snus has reduced smoking to a minimum, the remaining smokers seem to prefer e-cigarettes to snus if they should quit smoking. This indicates that availability of several nicotine alternatives might increase the likelihood of a future product replacement within the small group of remaining smokers.

## Introduction

In a European context, the Norwegian tobacco market is unique. While banned in the European Union, around 50 percent of the tobacco is consumed in the form of snus, a smokeless oral tobacco product. The corresponding percentage at the turn of the millennium was five.^1^ According to the Norwegian Smoking Habit Survey, use of snus among adults has been more widespread than smoking since 2018, and in 2021, current (daily or occasional) use of snus was reported by 25 percent among men and 10 percent among women. The corresponding figures for current snus use in 2000 was 12 percent among men and one percent among women.^2,3^

In comparison, 17 percent among men and 14 percent among women reported current cigarette smoking in 2021. This is less than half of what was reported in 2000 (42 percent among both men and women).^2^

In contrast, use of e-cigarettes or vaporizers has been rare. According to Norwegian Smoking Habit Survey, around three percent of adults used e-cigarettes daily or occasionally in the period 2015 to 2021, with little variation over years. Among never-smokers and never-snus users, the percentages of current use of e-cigarettes in this period were 0.5 and 2.3. Current e-cigarette use was most prevalent among daily smokers (12 percent) and occasional snus users (10 percent).^4^ In comparison, three percent of ever users of tobacco reported current use of nicotine replacement therapy products (NRT) in the same period.

The shift from cigarettes to snus are likely caused by three mechanisms. First, snus has long been the most frequently preferred smoking cessation product for both women and men.^5^ Second, a segment of smokers without intentions to quit have used snus instead of cigarettes in places where smoking is banned, or to reduce cigarette consumption.^6^ Third, young people susceptible to initiating tobacco use may have chosen snus instead of cigarettes.^7^ This is also the case in Sweden, the only country in the European Union where snus is legal and widespread.^8,9^

### Traits of Snus, E-cigarettes, and NRT Related to Openness to Quit Smoking

Snus, e-cigarettes, and NRT have all been subject to regulative, sociocultural, behavioral, product-specific, and epistemological differences that may affect smokers’ openness to use them as replacements for cigarettes if they want to quit smoking.

#### Snus

Snus has been on the market for generations and is well-known and easily accessible. Although daily snus use among never smokers has increased, a majority of existing snus users are still former smokers or dual users.^10^ Moreover, the product can be used indoors, comes in a variety of flavors and nicotine concentrations, and before the introduction of a plain packaging law in 2018, in cans of different sizes and graphical designs.^11^ The product variability provides snus with properties that can be used as markers of social distinction and user identity and snus use is not considered in the same negative way as smoking is.^12^ However, the low rate of adoption in the United States indicates that these conditions are not necessarily universal.^13^

However, smokers’ conversion to snus may be hampered by the health authorities’ opposition to use snus to quit smoking.^14^ From 2018, all snus cans in Norway have displayed the warning “Snus can damage your health and is addictive” and potential health hazards of snus use have received much media attention,^15,16^ partly because of two systematic reviews from the Norwegian Institute of Public Health that associated snus use with esophageal and pancreatic cancer, lethality after myocardial infarction and stroke, premature births, and type 2 diabetes among high-intensity consumers.^17,18^ Perhaps as a consequence, smokers perceive the risk of snus use to be around 80 percent the risk of smoking^19^—an overestimate inconsistent with assessments from expert groups.^20–23^ Swedish snus, while not being risk-free, has been assessed to be at the lower end of the tobacco products risk continuum,^24–26^ and the inverse trend for smoking and snus use in Norway and Sweden have been highlighted as a proof concept of tobacco harm reduction.^20,27,28^

#### E-cigarettes

Unlike snus, indoor use of e-cigarettes in public places is prohibited, availability is limited by a domestic ban on the sale of nicotine-containing e-juice, and most of the products are bought outside Norway.^29^ Vaping is also often portrayed as a technical task, which might be a barrier to potential switchers.^30^ Smokers must decide among a variety of devices, how to mix the e-juice, where to purchase nicotine and at what concentration, sort out any legal issues regarding purchase, choose among flavors, and set up and use their device safely. Converting from cigarette smoking to snus use involves no such technical hurdles.

Similar to snus, e-cigarettes have received much media attention, often concerning suspected negative health effects.^31^ Nevertheless, e-cigarettes have become the second most popular product used when attempting to quit smoking in Norway.^5^ Product variability, user motives and opportunities for involvement in vaping subcultures make e-cigarettes and vaping effective in identity communication^32^ and e-cigarettes have been characterized as occupying an ambiguous space between smoking and quitting.^33^ The usage ritual replicates the hand-to-mouth action and the sensory experience of smoking,^34^ and the uptake of nicotine into the bloodstream is comparable to cigarettes.^35^ Also, unique to e-cigarettes is that consumers are organized in active online vaping-dedicated discussion forums that provide technical assistance, offer emotional support, and encourage smokers to interact with vaping peers in vape shops. Furthermore, Norwegian smokers perceive e-cigarettes to be around 60 percent as harmful as smoking.^19^ According to a recent study, public opinion in Norway is less supportive of regulation of e-cigarettes compared to snus and cigarettes, and risk perceptions play a key role in forming public opinions on the strength of preventive regulations.^36^

#### Nicotine Replacement Therapy

In contrast to snus and e-cigarettes, which are subjected to an advertising ban, pharmaceutical companies run extensive advertising for nicotine replacement therapy products. In national guidelines for smoking cessation, use of NRT is recommended.

NRT has been on the Norwegian market since 1986. Unlike cigarettes, snus, and e-cigarettes, NRT products are not covered by the display ban introduced in 2010. And, similar to snus and e-cigarettes, smokers consider the health hazards of NRT to be lower compared to smoking (around 50 percent as harmful), but much higher than experts believe NRT to be.^19^

However, nicotine uptake from NRT is slower compared to vaping or snus use^37^ and the product is marketed as a drug intended for therapeutic use. In addition, the product portfolio is narrow and the flavors are fewer. Compared to snus and e-cigarettes, which have stand-alone recreational functions beyond their use in smoking cessation, recreational use of NRT products is uncommon and NRT does not have properties that can be used for identity formation, sociality, or social distinction. These conditions may reduce the potential of NRT to be an acceptable replacement for the many symbolic, behavioral, identity-formative, and sensory functions of cigarette smoking, which—to a certain extent—can be compensated by a transition to e-cigarettes or snus. Differences between the three products are summarized in Table 1.

**Table 1.** Differences in Regulative, Sociocultural, Behavioral, Product Specific and Epistemological Factors That Might Influence Smokers’ Openness to Replace Cigarettes With Snus, E-cigarettes, and NRT

	E-cigarettes	Snus	NRT
Regulative			
Advertising ban	Yes	Yes	No
Specific tax	No	Yes	No
Indoor use	No	Yes	Yes
Availability	Low	High	High
Flavor variability	Wide	Wide	Narrow
Plain packs	No	Yes	No
Display ban	Yes	Yes	No
Sociocultural			
Tradition	Short	Very long	Long
Use in population as a whole	1-2%	15%	-
Support from organized consumer groups	Yes	No	No
Used as marker of social distinction/identity	Yes	Yes	No
Behavioral			
Recreational use	Yes	Yes	No
Mimics smoking	Yes	No	No
Product specific			
Product diversity	Wide	Wide	Small
Potential for technical barriers	High	Low	Low
Pharmaceutical product	No	No	Yes
Speed of nicotine delivery	Fast	Moderate	Slow
Epistemological			
Recommended to quitters from authorities	No	No	Yes
Media attention	High	High	Low
Smokers’ perception of risk compared to cigarettes	60% of the risk	80% of the risk	50% of the risk

### Research Problems

Because snus has existed as a reduced-risk alternative to cigarettes for decades, snus may have reduced the market segment of health-conscious smokers that would have switched to e-cigarettes or NRT in a hypothetical absence of snus.^7^ At the same time, e-cigarettes or NRT may also appeal to a segment of smokers that has not been attracted to snus.

According to West et al. (2018) reach and effect are the two key components that will determine different products’ effect on smoking cessation at the population level^.38^ The overall aim of this study was therefore to examine Norwegian smokers’ openness, and thereby the potential reach, to use e-cigarettes, snus, and NRT in the event of quitting smoking. More specifically, we will:

I. Compare the openness to replace cigarettes with e-cigarettes, snus, and NRT in the event of quitting among (1) current smokers, (2) daily smokers, (3) occasional smokers, and (4) current smokers who have never used e-cigarettes or snus.
II. Examine the openness to use e-cigarettes, snus, and NRT among smokers with different socioeconomic characteristics, varying cigarette, e-cigarette, and snus use status and varying smoking histories and intentions to quit smoking.

## Methods

Questions about use of nicotine products were included in a weekly online omnibus survey in the period February 2019 to December 2021. The sampling procedure has been described in detail elsewhere.^39^ The study did not contain personally identifiable data, were in accordance with the Norwegian Health Research Act, and did not need approval from Regional Committees for Medical and Health Research Ethics.

### Measures

Smoking, snus, and e-cigarette use were assessed with three similar questions: “Which category best describes your current smoking or snus or e-cigarette use status?” Possible responses were: (1) *Current daily user*, (2) *Current occasional user*, *former daily user*, (3) *Current occasional user*, never daily user, (4) *Former daily user*, (5) *Former occasional user*, and (6) *Never user*. In the analyses we distinguish between *daily users* (1), *Occasional users* (2/3), *Former users* (4/5), and *Never users* (6). Use of NRT products was not measured in the survey.

Among a total of 18 617 ever smokers, 4073 were daily or occasional (current) smokers. They were asked, “Suppose you were to quit smoking altogether, would you consider using e-cigarettes, snus, or nicotine drugs instead of cigarettes?” Response options were: (1) *Yes, definitely*, (2) *Yes, probably*, (3) *Maybe*, (4) *No, probably not*, (5) *No, definitely not*, and (6) *I do not know*. In the analyses, we distinguish between smokers who were open (1/2), *not* open (4/5), and undecided (3/6) to use e-cigarettes, snus, or NRT in the event they should quit smoking.

All current smokers, snus, and e-cigarette users were asked which product they used, the first time they used nicotine. Possible answers were: (1) *Cigarette, cigar, cigarillo, or pipe*, (2) *Snus or chewing tobacco*, (3) *Nicotine gum, transdermal patch, or oral inhaler* and (4) *Electronic cigarettes*, and (5) *Have never used a nicotine product*. Smokers who chose alternative 5 (*n* = 95) were excluded from the analyses due to inconsistency.

Plans to quit smoking were assessed by the question “Do you envision smoking cessation?” with possible answers: (1) *Yes, within a month*, (2) *Yes within a year*, (3) *Yes, but I do not know when*, and (4) *No, I do not*.

Respondents’ socioeconomic position was measured by self-reported education and annual household income. Respondent’s education was categorized as *Primary* (9 years of compulsory education), *Secondary* (at least 3 years of high-school education), *Tertiary, lower* (Bachelor’s degree), and *Tertiary, higher* (Master’s degree or higher). Household income was measured by five categories: (1) *No reply/do not know*, (2) *Less than 500k NOK* (around 55k USD), (3) *500k to 1000k NOK*, (4) *1000k to 1500k NOK*, and (5) *Above 1500k NOK* (around 153k USD). Since we only had information on *household* income, we also included a control variable denoting the number of people living in the household. Table 2 presents the distribution of the independent variables.

**Table 2.** Sample Characteristics for Daily Smokers, Occasional Smokers, and Current Smokers (Daily and Occasional Smokers)

	Current smokers (*N* = 3978)		Daily smokers (*N* = 1881)		Occasional smokers (*N* = 2097)	
	Percent	*n*	Percent	*n*	Percent	*n*
Sex						
Men	48.3	1922	50.8	956	46.1	966
Women	51.7	2056	49.2	925	53.9	1131
Mean age	40.4	3978	47.7	1881	33.9	2097
Education						
Primary	10.7	423	13.5	252	8.2	171
Secondary	45.2	1787	44.4	828	45.9	959
Tertiary, lower	31.7	1254	32.8	612	30.7	642
Tertiary, higher	12.4	491	9.3	173	15.2	318
Household income (in NOK)						
No reply/ Do not know	15.4	612	13.3	251	17.2	361
less than 500 000	28.5	1135	31.4	590	26.0	545
500 000 to 1 000 000	34.9	1387	37.5	705	32.5	682
1 000 000 to 1 500 000	14.5	577	12.8	240	16.1	337
above 1 500 000	6.7	267	5.1	95	8.2	172
Smoking status						
Daily	47.3	1881	100.0	1881	0.0	0
Occasional	52.7	2097	0.0	0	100.0	2097
E-cigarette use status						
Daily	3.4	136	4.4	82	2.6	54
Occasional	10.5	419	10.0	188	11.0	231
Former	19.4	773	25.3	476	14.2	297
Never	66.6	2650	60.3	1135	72.2	1515
Snus use status						
Daily	21.3	847	7.3	138	33.8	709
Occasional	12.2	486	10.2	192	14.0	294
Former	11.2	447	12.0	226	10.5	221
Never	55.3	2198	70.4	1325	41.6	873
First used nicotine product						
Cigarettes, cigars, Cigarillos, or pipe	80.9	3217	93.1	1752	69.9	1465
Snus or chewing tobacco	15.7	623	4.7	88	25.5	535
NRT	1.7	66	1.4	26	1.9	40
E-cigarettes	1.8	72	0.8	15	2.7	57
Do you see yourself quitting smoking						
Yes, during the next 30 days	12.7	506	8.1	152	16.9	354
Yes, during the next year	12.9	515	15.8	298	10.3	217
Yes, but do not know when	49.6	1973	57.5	1082	42.5	891
No	24.7	984	18.6	349	30.3	635

### Analysis

Among all current smokers (*N* = 3978), 67 percent had never tried e-cigarettes, 55 percent had never tried snus and 40 percent had tried neither. To examine the openness to convert to e-cigarettes, snus, and NRT among smokers with different e-cigarette and snus use, we constructed a generalized structural equation model (gsem) in Stata 17.0^40^ with three discrete outcomes of openness (*Yes, definitely/yes, probably, Maybe/do not know,* and *No, definitely not/no, probably not* for e-cigarettes, snus, and NRT). Independent variables were a continuous measure of age (15–99) and dummy variables for sex, education, income, number of people in the household, planning to stop smoking, first product used, and cigarette, snus, and e-cigarette use status. Three thousand and nine hundred and thirty-seven smokers had information on all variables.

**Figure 1.**
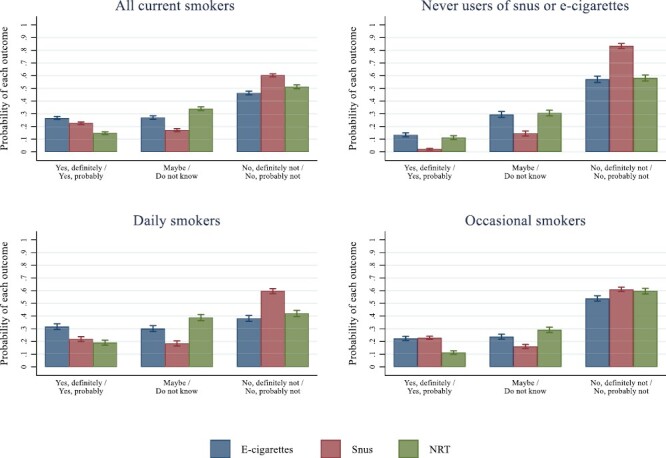
Predicted probabilities of being open to use e-cigarettes, snus, or NRT in the event of quitting smoking, by age, sex, education, household income, cigarette, e-cigarette or snus use status, plans to quit, and first nicotine or tobacco product use.

From this model, we calculated predicted probabilities of being open, not open, or undecided to use e-cigarettes/snus/NRT to quit smoking among smokers with different cigarette/e-cigarette/snus use status, and for each value of sex, education, income, planning to quit and first product used, and at selected values of age (25, …, 75). We chose to use a gsem model instead of three separate multinomial logistic regression models to be able to compare results across outcomes. Figure 1 and Supplementary Figures S1 and S2 (in Supplementary File 1) were made with the *coefplot*-package in Stata.^41^ Stata codes for the main model and all figures are provided in Supplementary File 2.

## Results

Among all current smokers, 47 percent were daily smokers, and 53 percent were occasional smokers (Table 2). Occasional smokers were on average 14 years younger than daily smokers. 81 percent of current smokers had initiated their nicotine use with cigarettes, while 16 percent responded that snus was the first product used. The corresponding percentage for e-cigarettes and NRT was two percent.

A much higher percentage among occasional smokers used snus currently (daily or occasionally) compared to daily smokers (48 vs. 18 percent), while the prevalence of current e-cigarette use was similar among occasional and daily smokers (14 percent). However, former e-cigarette use was higher among daily smokers compared to occasional smokers (25 vs. 14 percent).

Among all smokers, around ¼ believed they would be smoke-free within a year or earlier. The percentage who believed they would not quit smoking was almost twice as large among occasional smokers (30 percent) compared to daily smokers (19 percent).

**Figure 2.**
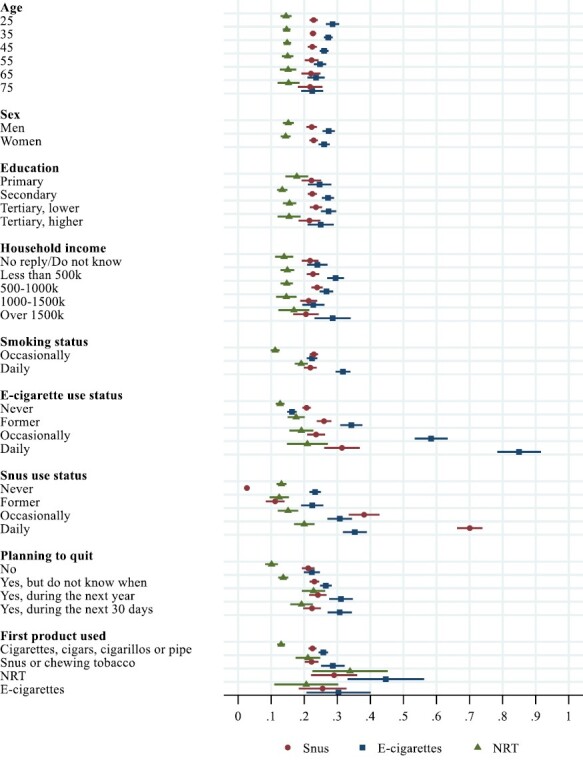
Predicted probability of being open, undecided, and *not* open to use e-cigarettes, snus, and NRT in the event of quitting smoking among all current smokers (top left), current smokers who had never used e-cigarettes or snus (top right), daily smokers (bottom left), and occasional smokers (bottom right).

The probabilities of being open, undecided, or not open to use e-cigarettes, snus, or NRT in the event of quitting smoking, calculated from the gsem model, are shown in Figure 2 (see Supplementary File 3 for coefficients and tests. All reported differences were statistically significant at the 5% level after Bonferroni correction). Among all current smokers, the probability of being open to use e-cigarettes (*Yes, definitely/yes, and probably)* in the event of quitting smoking was.27 (95% CI: 0.25 to 0.28, top left panel). The corresponding probabilities for snus and NRT were.23 (95% CI: 0.22 to 0.24) and.15 (95% CI: 0.14 to 0.16).

Snus was the product with the lowest probability of being undecided (“Maybe/Do not know”) (.17, 95% CI: 0.16 to 0.18) and the highest probability of *not* being open (“*No, definitely not/probably not*”) (.60, 95% CI: 0.59 to 0.61). NRT had the highest probability of being undecided (.34, 95% CI: 0.33 to 0.35).

Among daily smokers, the probability of being open to use e-cigarettes in the event of quitting smoking was higher (.32, 95% CI: 0.29 to 0.34) compared to using snus (.22, 95% CI: 0.20 to 0.24) or NRT (.19, 95% CI: 0.17 to 0.21). Among occasional smokers, there were no differences between e-cigarettes and snus (.22, 95% CI: 0.21 to 0.24 and.23, 95% CI: 0.22 to 0.24), but the probability of using NRT was significantly lower (.11, 95% CI: 0.10 to 0.13).

Among current smokers who had never used e-cigarettes or snus, the probability of being open to use snus was very low (.02, 95% CI: 0.02 to 0.03, top right panel) and the probability of *not* being open was very high (.83, 95% CI: 0.81 to 0.85). The probabilities of being open, undecided, or not open to use e-cigarettes or NRT in this group were similar.

Figure 1 shows the probability of being open to use e-cigarettes, snus, or NRT in the event of quitting smoking among current smokers, for all values of the independent variables.

The results show that there were no significant age gradients in the probabilities of being open to use of e-cigarettes, snus, or NRT, but the probability was generally lower for NRT compared to snus and e-cigarettes.

Compared to NRT, openness to use e-cigarettes was higher among respondents with secondary education or higher. Openness to use snus was higher compared to NRT among respondents with secondary or lower tertiary education. Smokers with low household income (less than 500k) were more open to use e-cigarettes (.29, 95% CI: 0.27 to 0.32) compared to both snus (.23, 95% CI: 0.21 to 0.25) and NRT (.15, 95% CI: 0.13 to 0.17).

The probability of being open to use e-cigarettes among daily e-cigarette users was .85 (95% CI: 0.78 to 0.92), while the probability of using snus among daily snus users was .70 (95% CI: 0.66 to 0.74). Among smokers who used e-cigarettes occasionally, the probability of being open to use e-cigarettes in the event of quitting smoking was .58 (95% CI: 0.53 to 0.63). The probability of using snus among smokers who used snus occasionally was .38 (95% CI: 0.33 to 0.43).

Never users of snus were less open to use snus in the event of quitting smoking (.03, 95% CI: 0.02 to 0.03) compared to the openness to use e-cigarettes among never users of e-cigarettes (.16, 95% CI: 0.15 to 0.18). Detailed results for all combinations of e-cigarette and snus use are provided in Supplementary Files 1 and 3.

The survey did not include a question on NRT use, but the probability of being open to use NRT among smokers who had never used e-cigarettes or snus was .13 in both cases (95% CI: 0.11 to 0.14 for e-cigarettes and .12–.15 for snus).

Smokers who had plans to quit smoking during the next month reported being more open to use e-cigarettes (.31, 95% CI: 0.27 to 0.34) compared to both snus (.22, 95% CI: 0.20 to 0.25) and NRT (.19, 95% CI: 0.16 to 0.23). For smokers with no plans to quit, there were no differences between snus and e-cigarettes (.22, 95% CI: 0.20 to 0.25 and.21, 95% CI: 0.19 to 0.23), but in both cases, the probabilities were higher compared to NRT (.10, 95% CI: 0.08 to 0.12).

Lastly, among smokers who had started their nicotine or tobacco use with cigarettes or other smoked tobacco products, e-cigarettes (.26, 95% CI: 0.24 to 0.27) and snus (.22, 95% CI: 0.21 to 0.24) had both higher probability of use in the event of quitting smoking compared to NRT (.13, 95% CI: 0.12 to 0.14).

## Discussion

In a snus-friendly norm climate where smokers traditionally have used snus as an alternative to cigarettes, a larger proportion of the remaining smokers were open to switch to e-cigarettes compared to snus, in the event they should stop smoking. The probability of using NRT in the event of quitting smoking was lower than both e-cigarettes and snus.

Moreover, the probability of *not* being open to use snus to quit smoking was much higher among snus users compared to e-cigarette users (.16 vs. .05 among daily users and .31 vs. .16 among occasional users, Supplementary File 1, Figure S2). This could indicate that a substantial segment of snus users would use a different product, or no product at all, in the event of quitting smoking.

In addition, the openness to replace cigarettes with e-cigarettes was relatively high among smokers with shorter education and lower household income (Figure 1). This could imply that e-cigarettes may play a role in reducing smoking-related health disparities. Besides tax hikes, few smoking-preventive interventions have an impact on socioeconomic differences.^42^ NRT was a less probable alternative, regardless of education or household income.

### Openness Among Naïve Users of E-cigarettes

The relative difference in openness was especially large among smokers who had never used e-cigarettes or snus (.13 for e-cigarettes, .11 for NRT, and .02 for snus, Figure 2). Added together, the probability of being undecided or positive to replace cigarettes with e-cigarettes among current smokers with no prior experience with e-cigarettes was .43. This indicates that even in a snus-rife society, where nicotine-containing e-cigarettes still are not available for sale, e-cigarettes are a potential reduced risk alternative to a substantial segment of the remaining smokers and equally attractive as NRT (.42), a product that has been on the market for decades and is actively encouraged by the health authorities. The corresponding probability for snus was .17, which might indicate that the future smoking cessation potential of snus may be limited.

### Openness Among Smokers Not Intending to Quit

Much of the population-based research on smoking cessation is focused on cigarette smokers who are already motivated to try to quit smoking.^43–45^ However, cigarette smokers not planning to quit smoking tend to have higher nicotine dependence, lower quitting self-efficacy, and be of lower socioeconomic status (SES) compared to smokers who do plan to quit.^46^ Some studies suggest that offering unmotivated smokers access to e-cigarettes might motivate them to quit.^47–50^

In our study, 25 percent did not see themselves quitting smoking (Table 2). Nevertheless, the probability of being open to use e-cigarettes or snus in the event of quitting in this group was .22 for e-cigarettes and .21 for snus. The corresponding probability for NRT was .10 (Figure 1). These findings underline the need to consider smokers who are not planning to quit when evaluating the risk-benefit potential of different products used for smoking cessation.

### Openness Among Former Users of E-cigarettes and Snus

Smokers who were former users of e-cigarettes were more positive to use e-cigarettes to quit smoking (.34) compared to using snus among former snus users (.11) (Figure 1). At the same time, the percentage among daily smokers having discontinued their use of e-cigarettes was twice as high as the proportion having stopped using snus (25 vs. 12 percent, Table 2). This may indicate that the importance of e-cigarettes as a permanent substitute for cigarettes could be weaker than what is the case for snus and may be related to product maladaptation.^30^ Research has shown that switching from smoking to vaping is a process of experimentation to find the right combination of device, nicotine, and flavor, which requires persistence and monetary investment. Relapse to smoking seems to be more common among ex-smokers vaping infrequently or using less advanced devices.^51^ Moreover, switching to e-cigarettes is associated with coughing and dry throat,^52^ often related to inadequate vaping technique or the mixing ratio of propylene glycol and glycerol in the e-juice.^53^ A qualitative study in the United Kingdom argued that smoking lapse is perceived qualitatively differently when using e-cigarettes compared to not using e-cigarettes to quit. When using e-cigarettes, lapses were perceived as more acceptable.^54^ It may be that snus provides fewer adaptive challenges and less discontinuation compared to e-cigarettes.

### Comparison to NRT

Overall, smokers were more open to use e-cigarettes and snus compared to NRT if they ever should stop smoking (.27 for e-cigarettes, .23 for snus, and .15 for NRT, Figure 2). However, this difference must be interpreted in light of the relatively large proportion of smokers who currently use e-cigarettes and snus (14 and 34 percent, respectively, Table 2). It is in these groups we find the highest likelihood of considering product substitution (Figure 1 and Supplementary File 1, Figure S1). As a therapeutic drug, use of NRT most typically occurs when smokers are in a quitting phase. The survey did not question NRT use, but when comparing openness to use NRT, snus, and e-cigarettes among never users of e-cigarettes or snus, we find that the probability of using NRT was slightly lower compared to e-cigarettes among never users of e-cigarettes (.13 vs. .16), but substantially higher compared to snus among never users of snus (.13 vs. .03, Figure 1), which suggest that NRT may still play a role in smoking cessation.

### Limitations

There are several limitations of this study. First, our outcome is measured with a non-validated instrument. A statement from smokers about preferences for alternative nicotine products in a future imaginary, and maybe irrelevant, situation, will necessarily have to be considered as a non-binding statement. It is neither a prediction, much less an intention. These statements are based on the existing knowledge and beliefs smokers have about alternative nicotine products and may be inconsistent with medical consensus.

Second, self-reported data on socially deviant behavior like smoking (and to a lesser degree vaping and snus use) may have led to misclassification of product use status due to social desirability bias. Also, because of the large variation between e-cigarette products, respondents may have different products in mind when indicating their openness to use e-cigarettes.

The strength of the study is the large sample of vapers and snus users, the consistent wording of the questions measuring smoking, vaping and snus use, and openness to replace cigarettes with other products.

With regards to representativeness of smoking and snus use, we compared figures from the present dataset with a comparable population (aged 16–79 years in the years 2015–2019) from a nationally representative survey conducted annually by Statistics Norway. The prevalence of current snus use was 15 percent in both surveys. The corresponding figures for current smoking were 16 percent in the current survey and 19 percent in the nationally representative survey.

## Supplementary Material

A Contributorship Form detailing each author’s specific involvement with this content, as well as any supplementary data, are available online at https://academic.oup.com/ntr.

ntad113_suppl_Supplementary_Materials

## Data Availability

Data were collected by Ipsos on behalf of the Norwegian Institute of Public Health and are available upon request.
