# Supplementary material for: How do Smokers in a Snus-Prevalent Society Consider E-cigarettes, Snus, and Nicotine Replacement Therapy Products as Relevant Replacements for Cigarettes in the Event They Should Stop Smoking?
Source: Nicotine Tob Res. 2023 Jul 6;25(11):1753–61. doi: 10.1093/ntr/ntad113 (PMC10475606; doi:10.1093/ntr/ntad113)
Supplement: ntad113_suppl_Supplementary_Materials [file ntad113_suppl_supplementary_materials.zip › Supplementary file 3 - Estimates_V2.docx]

# Estimates for Figure 1 and 2, and Supplementary Figure S1 and S2

**Figure 1. Predicted probability of being open, undecided and *not* open to use e-cigarettes, snus and NRT in the event of quitting smoking among all current smokers, current smokers who had never used e-cigarettes or snus, daily smokers and occasional smokers**

|  |  |  | **Margin** | **Delta-method std. err.** | **z** | **P>\|z\|** | **[95% conf. interval]** | | **Pairwise comparison*** |
| --- | --- | --- | --- | --- | --- | --- | --- | --- | --- |
| Alls smokers | Open to use e-cigarettes | Yes, definitely / Yes, probably | 0.267 | 0.006 | 42.640 | 0.000 | 0.254 | 0.279 | B |
|  |  | Maybe / Do not know | 0.270 | 0.007 | 38.780 | 0.000 | 0.256 | 0.283 | B |
|  |  | No, definitely not / No, probably not | 0.464 | 0.007 | 63.530 | 0.000 | 0.449 | 0.478 |  |
|  | Open to use snus | Yes, definitely / Yes, probably | 0.226 | 0.005 | 45.740 | 0.000 | 0.216 | 0.236 |  |
|  |  | Maybe / Do not know | 0.171 | 0.006 | 29.220 | 0.000 | 0.160 | 0.183 | A |
|  |  | No, definitely not / No, probably not | 0.603 | 0.006 | 98.990 | 0.000 | 0.591 | 0.615 |  |
|  | Open to use NRT | Yes, definitely / Yes, probably | 0.148 | 0.005 | 26.920 | 0.000 | 0.137 | 0.158 | A |
|  |  | Maybe / Do not know | 0.340 | 0.007 | 45.850 | 0.000 | 0.325 | 0.354 |  |
|  |  | No, definitely not / No, probably not | 0.513 | 0.008 | 67.030 | 0.000 | 0.498 | 0.528 |  |
| Never users of e-cigarettes ort snus | Open to use e-cigarettes | Yes, definitely / Yes, probably | 0.134 | 0.008 | 16.640 | 0.000 | 0.118 | 0.150 |  |
|  |  | Maybe / Do not know | 0.294 | 0.012 | 24.560 | 0.000 | 0.271 | 0.318 |  |
|  |  | No, definitely not / No, probably not | 0.572 | 0.013 | 45.010 | 0.000 | 0.547 | 0.597 |  |
|  | Open to use snus | Yes, definitely / Yes, probably | 0.021 | 0.003 | 6.690 | 0.000 | 0.015 | 0.028 |  |
|  |  | Maybe / Do not know | 0.145 | 0.010 | 14.840 | 0.000 | 0.126 | 0.164 |  |
|  |  | No, definitely not / No, probably not | 0.834 | 0.010 | 81.600 | 0.000 | 0.814 | 0.854 |  |
|  | Open to use NRT | Yes, definitely / Yes, probably | 0.112 | 0.008 | 14.640 | 0.000 | 0.097 | 0.127 |  |
|  |  | Maybe / Do not know | 0.306 | 0.011 | 26.870 | 0.000 | 0.284 | 0.328 |  |
|  |  | No, definitely not / No, probably not | 0.582 | 0.012 | 48.070 | 0.000 | 0.558 | 0.606 |  |
| Daily smokers | Open to use e-cigarettes | Yes, definitely / Yes, probably | 0.317 | 0.011 | 27.970 | 0.000 | 0.295 | 0.339 | B |
|  |  | Maybe / Do not know | 0.301 | 0.012 | 25.910 | 0.000 | 0.278 | 0.324 | B |
|  |  | No, definitely not / No, probably not | 0.382 | 0.012 | 32.420 | 0.000 | 0.359 | 0.405 | C |
|  | Open to use snus | Yes, definitely / Yes, probably | 0.219 | 0.010 | 22.110 | 0.000 | 0.199 | 0.238 | A |
|  |  | Maybe / Do not know | 0.185 | 0.010 | 17.950 | 0.000 | 0.165 | 0.205 | A |
|  |  | No, definitely not / No, probably not | 0.596 | 0.010 | 58.810 | 0.000 | 0.577 | 0.616 |  |
|  | Open to use NRT | Yes, definitely / Yes, probably | 0.191 | 0.010 | 19.100 | 0.000 | 0.171 | 0.211 | A |
|  |  | Maybe / Do not know | 0.388 | 0.012 | 31.510 | 0.000 | 0.364 | 0.412 | C |
|  |  | No, definitely not / No, probably not | 0.421 | 0.012 | 34.220 | 0.000 | 0.397 | 0.445 | C |
| Occasional smokers | Open to use e-cigarettes | Yes, definitely / Yes, probably | 0.224 | 0.008 | 27.040 | 0.000 | 0.207 | 0.240 | A |
|  |  | Maybe / Do not know | 0.238 | 0.010 | 23.660 | 0.000 | 0.218 | 0.257 | A |
|  |  | No, definitely not / No, probably not | 0.539 | 0.011 | 49.960 | 0.000 | 0.518 | 0.560 |  |
|  | Open to use snus | Yes, definitely / Yes, probably | 0.229 | 0.006 | 35.730 | 0.000 | 0.217 | 0.242 | A |
|  |  | Maybe / Do not know | 0.161 | 0.008 | 19.560 | 0.000 | 0.145 | 0.177 |  |
|  |  | No, definitely not / No, probably not | 0.610 | 0.009 | 69.950 | 0.000 | 0.593 | 0.627 | B |
|  | Open to use NRT | Yes, definitely / Yes, probably | 0.112 | 0.007 | 16.270 | 0.000 | 0.099 | 0.126 |  |
|  |  | Maybe / Do not know | 0.292 | 0.011 | 27.660 | 0.000 | 0.271 | 0.312 |  |
|  |  | No, definitely not / No, probably not | 0.596 | 0.011 | 53.040 | 0.000 | 0.574 | 0.618 | B |

*Margins sharing a letter in the group label are not significantly different at the 5% level. Withing smoking status categories (All smokers / Never users of e-cigarettes ort snus / Daily smokers / Occasional smokers). Bonferroni method.

**Figure 2. Predicted probabilities of being open to use e-cigarettes, snus or NRT in the event of quitting smoking, by age, sex, education, household income, cigarette/e-cigarette/snus use status, plans to quit and first nicotine or tobacco product used**

|  | **Open to use e-cigarettes** | | | | | |  | **Open to use snus** | | | | | |  | **Open to use NRT** | | | | | |  |
| --- | --- | --- | --- | --- | --- | --- | --- | --- | --- | --- | --- | --- | --- | --- | --- | --- | --- | --- | --- | --- | --- |
|  | **Margin** | **Delta-method std. err.** | **z** | **P>\|z\|** | **[95% conf. interval]** | | **Pairwise comparison*** | **Margin** | **Delta-method std. err.** | **z** | **P>\|z\|** | **[95% conf. interval]** | | **Pairwise comparison*** | **Margin** | **Delta-method std. err.** | **z** | **P>\|z\|** | **[95% conf. interval]** | | **Pairwise comparison*** |
| **Age** |  |  |  |  |  |  |  |  |  |  |  |  |  |  |  |  |  |  |  |  |  |
| 25 | 0.286 | 0.010 | 27.750 | 0.000 | 0.265 | 0.306 | C | 0.228 | 0.006 | 36.220 | 0.000 | 0.216 | 0.241 | C | 0.145 | 0.008 | 17.220 | 0.000 | 0.129 | 0.162 | A |
| 35 | 0.273 | 0.007 | 39.860 | 0.000 | 0.259 | 0.286 | C | 0.227 | 0.005 | 44.790 | 0.000 | 0.217 | 0.236 | C | 0.147 | 0.006 | 25.050 | 0.000 | 0.135 | 0.158 | A |
| 45 | 0.260 | 0.007 | 38.440 | 0.000 | 0.247 | 0.273 | C | 0.224 | 0.007 | 31.320 | 0.000 | 0.210 | 0.239 | C | 0.148 | 0.006 | 24.910 | 0.000 | 0.137 | 0.160 | A |
| 55 | 0.248 | 0.010 | 25.800 | 0.000 | 0.229 | 0.267 | C | 0.222 | 0.011 | 20.680 | 0.000 | 0.201 | 0.243 | C | 0.150 | 0.009 | 17.140 | 0.000 | 0.133 | 0.167 | A |
| 65 | 0.236 | 0.013 | 17.760 | 0.000 | 0.210 | 0.262 | C | 0.220 | 0.015 | 14.950 | 0.000 | 0.191 | 0.249 | BC | 0.151 | 0.013 | 11.980 | 0.000 | 0.127 | 0.176 | A |
| 75 | 0.224 | 0.017 | 13.200 | 0.000 | 0.191 | 0.257 | BC | 0.218 | 0.019 | 11.580 | 0.000 | 0.181 | 0.255 | ABC | 0.153 | 0.017 | 9.020 | 0.000 | 0.119 | 0.186 | AB |
| **Sex** |  |  |  |  |  |  |  |  |  |  |  |  |  |  |  |  |  |  |  |  |  |
| Men | 0.274 | 0.010 | 28.830 | 0.000 | 0.255 | 0.293 | C | 0.223 | 0.008 | 27.320 | 0.000 | 0.207 | 0.239 | B | 0.152 | 0.008 | 18.060 | 0.000 | 0.135 | 0.168 | A |
| Women | 0.260 | 0.009 | 30.050 | 0.000 | 0.243 | 0.277 | C | 0.229 | 0.007 | 34.850 | 0.000 | 0.216 | 0.241 | B | 0.144 | 0.008 | 19.160 | 0.000 | 0.129 | 0.158 | A |
| **Education** |  |  |  |  |  |  |  |  |  |  |  |  |  |  |  |  |  |  |  |  |  |
| Tertiary, higher | 0.250 | 0.020 | 12.330 | 0.000 | 0.210 | 0.289 | DE | 0.216 | 0.017 | 12.820 | 0.000 | 0.183 | 0.249 | BCDE | 0.154 | 0.018 | 8.680 | 0.000 | 0.119 | 0.189 | ABC |
| Tertiary, lower | 0.274 | 0.012 | 23.390 | 0.000 | 0.251 | 0.296 | E | 0.236 | 0.009 | 25.000 | 0.000 | 0.217 | 0.254 | DE | 0.156 | 0.010 | 15.250 | 0.000 | 0.136 | 0.176 | AB |
| Secondary | 0.272 | 0.009 | 29.060 | 0.000 | 0.254 | 0.290 | E | 0.224 | 0.007 | 31.600 | 0.000 | 0.211 | 0.238 | DE | 0.133 | 0.008 | 16.820 | 0.000 | 0.118 | 0.149 | A |
| Primary | 0.247 | 0.018 | 13.640 | 0.000 | 0.211 | 0.282 | DE | 0.222 | 0.015 | 14.660 | 0.000 | 0.192 | 0.251 | CDE | 0.178 | 0.018 | 10.080 | 0.000 | 0.143 | 0.212 | ABCD |
| **Income** |  |  |  |  |  |  |  |  |  |  |  |  |  |  |  |  |  |  |  |  |  |
| No reply / Do not know | 0.239 | 0.016 | 15.140 | 0.000 | 0.208 | 0.270 | BCD | 0.218 | 0.013 | 16.970 | 0.000 | 0.193 | 0.243 | BC | 0.139 | 0.014 | 9.900 | 0.000 | 0.112 | 0.167 | A |
| Less than 500k | 0.295 | 0.013 | 22.800 | 0.000 | 0.270 | 0.320 | D | 0.226 | 0.010 | 23.360 | 0.000 | 0.207 | 0.245 | BC | 0.149 | 0.011 | 14.040 | 0.000 | 0.128 | 0.170 | A |
| 500-1000k | 0.267 | 0.011 | 25.170 | 0.000 | 0.246 | 0.288 | CD | 0.239 | 0.009 | 27.180 | 0.000 | 0.222 | 0.256 | BC | 0.147 | 0.010 | 15.430 | 0.000 | 0.129 | 0.166 | A |
| 1000-1500k | 0.227 | 0.017 | 13.270 | 0.000 | 0.194 | 0.261 | BCD | 0.213 | 0.013 | 16.260 | 0.000 | 0.188 | 0.239 | BC | 0.146 | 0.016 | 9.250 | 0.000 | 0.115 | 0.177 | A |
| Over 1500k | 0.286 | 0.028 | 10.240 | 0.000 | 0.231 | 0.341 | CD | 0.205 | 0.020 | 10.460 | 0.000 | 0.167 | 0.244 | ABC | 0.169 | 0.024 | 7.040 | 0.000 | 0.122 | 0.216 | AB |
| **Planning to quit smoking** |  |  |  |  |  |  |  |  |  |  |  |  |  |  |  |  |  |  |  |  |  |
| No | 0.223 | 0.012 | 18.010 | 0.000 | 0.199 | 0.247 | CD | 0.212 | 0.010 | 21.800 | 0.000 | 0.193 | 0.231 | C | 0.101 | 0.010 | 10.420 | 0.000 | 0.082 | 0.120 | A |
| Yes, but do not know when | 0.265 | 0.009 | 29.510 | 0.000 | 0.248 | 0.283 | DEF | 0.231 | 0.007 | 31.300 | 0.000 | 0.216 | 0.245 | CD | 0.137 | 0.008 | 17.700 | 0.000 | 0.121 | 0.152 | AB |
| Yes, during the next year | 0.311 | 0.018 | 17.160 | 0.000 | 0.276 | 0.347 | F | 0.241 | 0.013 | 18.010 | 0.000 | 0.215 | 0.268 | CDEF | 0.228 | 0.018 | 12.730 | 0.000 | 0.193 | 0.263 | CDE |
| Yes, during the next 30 days | 0.307 | 0.019 | 16.370 | 0.000 | 0.271 | 0.344 | EF | 0.224 | 0.014 | 16.460 | 0.000 | 0.197 | 0.250 | CD | 0.192 | 0.018 | 10.920 | 0.000 | 0.157 | 0.226 | BC |
| **First product used** |  |  |  |  |  |  |  |  |  |  |  |  |  |  |  |  |  |  |  |  |  |
| Cigarettes, cigars/cigarillos or pipe | 0.258 | 0.007 | 34.970 | 0.000 | 0.243 | 0.272 | BC | 0.225 | 0.006 | 36.640 | 0.000 | 0.213 | 0.237 | B | 0.130 | 0.006 | 21.880 | 0.000 | 0.119 | 0.142 | A |
| Snus or chewing tobacco | 0.287 | 0.018 | 15.780 | 0.000 | 0.251 | 0.322 | BC | 0.222 | 0.011 | 21.080 | 0.000 | 0.202 | 0.243 | B | 0.211 | 0.019 | 11.070 | 0.000 | 0.174 | 0.249 | B |
| NRT | 0.447 | 0.059 | 7.550 | 0.000 | 0.331 | 0.563 | C | 0.290 | 0.036 | 8.140 | 0.000 | 0.220 | 0.360 | BC | 0.338 | 0.058 | 5.830 | 0.000 | 0.225 | 0.452 | BC |
| E-cigarettes | 0.304 | 0.050 | 6.130 | 0.000 | 0.207 | 0.401 | BC | 0.256 | 0.037 | 6.910 | 0.000 | 0.183 | 0.328 | ABC | 0.206 | 0.049 | 4.180 | 0.000 | 0.110 | 0.303 | ABC |
| **Smoking status** |  |  |  |  |  |  |  |  |  |  |  |  |  |  |  |  |  |  |  |  |  |
| Occasionally | 0.224 | 0.008 | 27.040 | 0.000 | 0.207 | 0.240 | AB | 0.229 | 0.006 | 35.730 | 0.000 | 0.217 | 0.242 | B | 0.112 | 0.007 | 16.270 | 0.000 | 0.099 | 0.126 |  |
| Daily | 0.317 | 0.011 | 27.970 | 0.000 | 0.295 | 0.339 |  | 0.219 | 0.010 | 22.110 | 0.000 | 0.199 | 0.238 | AB | 0.191 | 0.010 | 19.100 | 0.000 | 0.171 | 0.211 | A |
| **Snus use status** |  |  |  |  |  |  |  |  |  |  |  |  |  |  |  |  |  |  |  |  |  |
| Never | 0.233 | 0.009 | 26.130 | 0.000 | 0.215 | 0.250 | C | 0.027 | 0.004 | 7.090 | 0.000 | 0.019 | 0.034 |  | 0.131 | 0.008 | 17.190 | 0.000 | 0.116 | 0.146 | A |
| Former | 0.224 | 0.017 | 13.060 | 0.000 | 0.191 | 0.258 | BCD | 0.112 | 0.015 | 7.730 | 0.000 | 0.084 | 0.140 | A | 0.124 | 0.015 | 8.350 | 0.000 | 0.095 | 0.154 | A |
| Occasionally | 0.307 | 0.019 | 16.170 | 0.000 | 0.270 | 0.345 | DE | 0.381 | 0.024 | 16.090 | 0.000 | 0.335 | 0.428 | E | 0.151 | 0.016 | 9.530 | 0.000 | 0.120 | 0.182 | AB |
| Daily | 0.353 | 0.019 | 19.070 | 0.000 | 0.317 | 0.389 | E | 0.701 | 0.020 | 35.680 | 0.000 | 0.662 | 0.739 |  | 0.200 | 0.016 | 12.660 | 0.000 | 0.169 | 0.231 | BC |
| **E-cigarette use status** |  |  |  |  |  |  |  |  |  |  |  |  |  |  |  |  |  |  |  |  |  |
| Never | 0.163 | 0.007 | 22.040 | 0.000 | 0.148 | 0.177 | B | 0.207 | 0.006 | 32.260 | 0.000 | 0.194 | 0.220 | C | 0.127 | 0.007 | 18.940 | 0.000 | 0.114 | 0.140 | A |
| Former | 0.342 | 0.017 | 19.980 | 0.000 | 0.309 | 0.376 | F | 0.260 | 0.011 | 22.880 | 0.000 | 0.238 | 0.282 | DE | 0.176 | 0.013 | 13.150 | 0.000 | 0.149 | 0.202 | ABC |
| Occasionally | 0.584 | 0.025 | 22.930 | 0.000 | 0.534 | 0.634 |  | 0.236 | 0.014 | 17.130 | 0.000 | 0.209 | 0.263 | CDE | 0.192 | 0.019 | 10.340 | 0.000 | 0.155 | 0.228 | ABCD |
| Daily | 0.850 | 0.034 | 25.220 | 0.000 | 0.784 | 0.916 |  | 0.314 | 0.027 | 11.460 | 0.000 | 0.260 | 0.368 | EF | 0.210 | 0.032 | 6.630 | 0.000 | 0.148 | 0.271 | ABCDE |

*Margins sharing a letter in the group label are not significantly different at the 5% level. Within independent variables and across dependent variables (Open to use e-cigarettes / Open to use snus / Open to use NRT). Bonferroni method.

**Figure S1. Predicted probability of being open to use e-cigarettes or snus in the event of quitting smoking, by combinations of e-cigarette and snus use status (Supplementary file 1)**

| **Open to use e-cigarettes** | | | | | | | | | | | | | | | |  |
| --- | --- | --- | --- | --- | --- | --- | --- | --- | --- | --- | --- | --- | --- | --- | --- | --- |
| **E-cigarette use status** | **Snus use status** | | **Margin** | | **Delta-method std. err.** | | **z** | | **P>\|z\|** | | **[95% conf. interval]** | | | **Pairwise comparison*** | |  |
| Never | Never | | 0.134 | | 0.008 | | 16.640 | | 0.000 | | 0.118 | | 0.150 | EF | |  |
| Never | Former | | 0.126 | | 0.014 | | 8.740 | | 0.000 | | 0.098 | | 0.154 | DEF | |  |
| Never | Occasionally | | 0.199 | | 0.019 | | 10.680 | | 0.000 | | 0.163 | | 0.236 | FGH | |  |
| Never | Daily | | 0.241 | | 0.019 | | 12.500 | | 0.000 | | 0.203 | | 0.278 | GHI | |  |
| **Never (mean)** |  | | **0.163** | | **0.007** | | **22.040** | | **0.000** | | **0.148** | | **0.177** | **A** | |  |
| Former | Never | | 0.296 | | 0.019 | | 15.210 | | 0.000 | | 0.258 | | 0.335 | HIJ | |  |
| Former | Former | | 0.284 | | 0.028 | | 10.240 | | 0.000 | | 0.230 | | 0.338 | HIJ | |  |
| Former | Occasionally | | 0.400 | | 0.030 | | 13.250 | | 0.000 | | 0.341 | | 0.459 | JKLM | |  |
| Former | Daily | | 0.461 | | 0.028 | | 16.630 | | 0.000 | | 0.406 | | 0.515 | LM | |  |
| **Former (mean)** |  | | **0.342** | | **0.017** | | **19.980** | | **0.000** | | **0.309** | | **0.376** | **D** | |  |
| Occasional | Never | | 0.536 | | 0.029 | | 18.460 | | 0.000 | | 0.479 | | 0.593 | MNO | |  |
| Occasional | Former | | 0.528 | | 0.039 | | 13.560 | | 0.000 | | 0.452 | | 0.604 | MNO | |  |
| Occasional | Occasionally | | 0.641 | | 0.032 | | 19.950 | | 0.000 | | 0.578 | | 0.704 | NOP | |  |
| Occasional | Daily | | 0.701 | | 0.030 | | 23.470 | | 0.000 | | 0.643 | | 0.760 | PQ | |  |
| **Occasional (mean)** |  | | **0.584** | | **0.025** | | **22.930** | | **0.000** | | **0.534** | | **0.634** |  | |  |
| Daily | Never | | 0.826 | | 0.038 | | 21.690 | | 0.000 | | 0.751 | | 0.900 | PQR | |  |
| Daily | Former | | 0.822 | | 0.043 | | 19.300 | | 0.000 | | 0.739 | | 0.906 | PQRS | |  |
| Daily | Occasionally | | 0.880 | | 0.031 | | 28.690 | | 0.000 | | 0.819 | | 0.940 | RS | |  |
| Daily | Daily | | 0.906 | | 0.025 | | 35.920 | | 0.000 | | 0.857 | | 0.956 | S | |  |
| **Daily (mean)** |  | | **0.850** | | **0.034** | | **25.220** | | **0.000** | | **0.784** | | **0.916** |  | |  |
| **Open to use snus** | | | | | | | | | | | | | | | | |
| **Snus use status** | **E-cigarette use status** | **Margin** | | **Delta-method std. err.** | | **z** | | **P>\|z\|** | | **[95% conf. interval]** | | | | | **Pairwise comparison*** | |
| Never | Never | 0.021 | | 0.003 | | 6.690 | | 0.000 | | 0.015 | | 0.028 | | | A | |
| Never | Former | 0.037 | | 0.006 | | 5.870 | | 0.000 | | 0.025 | | 0.049 | | | ABC | |
| Never | Occasionally | 0.029 | | 0.006 | | 5.000 | | 0.000 | | 0.018 | | 0.040 | | | AB | |
| Never | Daily | 0.058 | | 0.016 | | 3.620 | | 0.000 | | 0.026 | | 0.089 | | | ABCD | |
| **Never (mean)** |  | **0.027** | | **0.004** | | **7.090** | | **0.000** | | **0.019** | | **0.034** | | |  | |
| Former | Never | 0.093 | | 0.013 | | 6.900 | | 0.000 | | 0.067 | | 0.119 | | | CDE | |
| Former | Former | 0.151 | | 0.022 | | 6.840 | | 0.000 | | 0.108 | | 0.194 | | | DEFG | |
| Former | Occasionally | 0.122 | | 0.021 | | 5.720 | | 0.000 | | 0.080 | | 0.163 | | | CDEF | |
| Former | Daily | 0.226 | | 0.051 | | 4.420 | | 0.000 | | 0.126 | | 0.326 | | | B EFGHIJK | |
| **Former (mean)** |  | **0.112** | | **0.015** | | **7.730** | | **0.000** | | **0.084** | | **0.140** | | | **A** | |
| Occasional | Never | 0.340 | | 0.025 | | 13.480 | | 0.000 | | 0.291 | | 0.390 | | | IJ L | |
| Occasional | Former | 0.468 | | 0.036 | | 12.960 | | 0.000 | | 0.397 | | 0.539 | | | K MN | |
| Occasional | Occasionally | 0.410 | | 0.039 | | 10.560 | | 0.000 | | 0.334 | | 0.486 | | | JKLM | |
| Occasional | Daily | 0.607 | | 0.068 | | 8.890 | | 0.000 | | 0.473 | | 0.740 | | | MNOP | |
| **Occasional (mean)** |  | **0.381** | | **0.024** | | **16.090** | | **0.000** | | **0.335** | | **0.428** | | | **D** | |
| Daily | Never | 0.668 | | 0.022 | | 29.730 | | 0.000 | | 0.624 | | 0.712 | | | OP | |
| Daily | Former | 0.774 | | 0.024 | | 31.640 | | 0.000 | | 0.726 | | 0.822 | | | PQR | |
| Daily | Occasionally | 0.730 | | 0.033 | | 21.980 | | 0.000 | | 0.665 | | 0.795 | | | PQR | |
| Daily | Daily | 0.856 | | 0.036 | | 24.010 | | 0.000 | | 0.786 | | 0.926 | | | QRS | |
| **Daily (mean)** |  | **0.701** | | **0.020** | | **35.680** | | **0.000** | | **0.662** | | **0.739** | | |  | |

*Margins sharing a letter in the group label are not significantly different at the 5% level. Within and between dependent variables (Open to use e-cigarettes / Open to use sus). Mean values were tested separately. Bonferroni method.

**Figure S2. Predicted probability of being *not* open to use e-cigarettes or snus in the event of quitting smoking, by combinations of e-cigarette and snus use status (Supplementary file 1)**

| **Not open to use e-cigarettes** | | | | | | | | | | | | | | | | |
| --- | --- | --- | --- | --- | --- | --- | --- | --- | --- | --- | --- | --- | --- | --- | --- | --- |
| **E-cigarette use status** | **Snus use status** | | | **Margin** | | **Delta-method std. err.** | | **z** | | **P>\|z\|** | | **[95% conf. interval]** | | **Pairwise comparison*** | | |
| Never | Never | | | 0.572 | | 0.013 | | 45.010 | | 0.000 | | 0.547 | 0.597 | MNO | | |
| Never | Former | | | 0.605 | | 0.026 | | 23.230 | | 0.000 | | 0.554 | 0.657 | NO | | |
| Never | Occasionally | | | 0.504 | | 0.027 | | 18.650 | | 0.000 | | 0.451 | 0.557 | LMN | | |
| Never | Daily | | | 0.498 | | 0.022 | | 22.810 | | 0.000 | | 0.455 | 0.541 | LMN | | |
| **Never (mean)** |  | | | **0.552** | | **0.010** | | **57.040** | | **0.000** | | **0.533** | **0.571** |  | | |
| Former | Never | | | 0.405 | | 0.021 | | 19.540 | | 0.000 | | 0.365 | 0.446 | JKL | | |
| Former | Former | | | 0.438 | | 0.030 | | 14.610 | | 0.000 | | 0.379 | 0.497 | KLM | | |
| Former | Occasionally | | | 0.326 | | 0.028 | | 11.790 | | 0.000 | | 0.272 | 0.381 | HIJK | | |
| Former | Daily | | | 0.309 | | 0.023 | | 13.190 | | 0.000 | | 0.263 | 0.354 | HIJ | | |
| **Former (mean)** |  | | | **0.379** | | **0.018** | | **21.260** | | **0.000** | | **0.344** | **0.414** | **B** | | |
| Occasional | Never | | | 0.180 | | 0.023 | | 7.870 | | 0.000 | | 0.135 | 0.225 | D FGH | | |
| Occasional | Former | | | 0.201 | | 0.029 | | 6.910 | | 0.000 | | 0.144 | 0.258 | CD FGHI | | |
| Occasional | Occasionally | | | 0.128 | | 0.020 | | 6.550 | | 0.000 | | 0.090 | 0.167 | CDE | | |
| Occasional | Daily | | | 0.115 | | 0.018 | | 6.370 | | 0.000 | | 0.079 | 0.150 | BCDE | | |
| **Occasional (mean)** |  | | | **0.162** | | **0.020** | | **8.030** | | **0.000** | | **0.123** | **0.202** | **A** | | |
| Daily | Never | | | 0.054 | | 0.023 | | 2.290 | | 0.022 | | 0.008 | 0.099 | ABC | | |
| Daily | Former | | | 0.061 | | 0.026 | | 2.300 | | 0.022 | | 0.009 | 0.112 | ABCD | | |
| Daily | Occasionally | | | 0.033 | | 0.015 | | 2.190 | | 0.028 | | 0.004 | 0.063 | AB | | |
| Daily | Daily | | | 0.028 | | 0.013 | | 2.160 | | 0.031 | | 0.003 | 0.054 | A | | |
| **Daily (mean)** |  | | | **0.046** | | **0.020** | | **2.290** | | **0.022** | | **0.007** | **0.086** |  | | |
| **Not open to use snus** | | | | | | | | | | | | | | | | |
| **Snus use status** | | **E-cigarette use status** | **Margin** | | **Delta-method std. err.** | | **z** | | **P>\|z\|** | | **[95% conf. interval]** | | | | **Pairwise comparison*** |  |
| Never | | Never | 0.834 | | 0.010 | | 81.600 | | 0.000 | | 0.814 | | 0.854 | | R |  |
| Never | | Former | 0.814 | | 0.017 | | 48.860 | | 0.000 | | 0.781 | | 0.846 | | P R |  |
| Never | | Occasionally | 0.827 | | 0.019 | | 42.710 | | 0.000 | | 0.789 | | 0.865 | | P R |  |
| Never | | Daily | 0.829 | | 0.033 | | 25.300 | | 0.000 | | 0.765 | | 0.893 | | QR |  |
| **Never (mean)** | |  | **0.829** | | **0.009** | | **88.400** | | **0.000** | | **0.810** | | **0.847** | |  |  |
| Former | | Never | 0.664 | | 0.024 | | 27.900 | | 0.000 | | 0.617 | | 0.711 | | O Q |  |
| Former | | Former | 0.612 | | 0.030 | | 20.280 | | 0.000 | | 0.553 | | 0.671 | | NO |  |
| Former | | Occasionally | 0.642 | | 0.034 | | 18.730 | | 0.000 | | 0.575 | | 0.709 | | NO Q |  |
| Former | | Daily | 0.601 | | 0.057 | | 10.590 | | 0.000 | | 0.490 | | 0.712 | | LMNOP |  |
| **Former (mean)** | |  | **0.649** | | **0.023** | | **28.610** | | **0.000** | | **0.605** | | **0.694** | |  |  |
| Occasional | | Never | 0.328 | | 0.025 | | 13.160 | | 0.000 | | 0.279 | | 0.376 | | IJK |  |
| Occasional | | Former | 0.257 | | 0.027 | | 9.600 | | 0.000 | | 0.204 | | 0.309 | | FGHI |  |
| Occasional | | Occasionally | 0.292 | | 0.031 | | 9.440 | | 0.000 | | 0.231 | | 0.353 | | GHIJK |  |
| Occasional | | Daily | 0.219 | | 0.044 | | 4.960 | | 0.000 | | 0.132 | | 0.305 | | C EFGHIJ |  |
| **Occasional (mean)** | |  | **0.307** | | **0.023** | | **13.560** | | **0.000** | | **0.263** | | **0.352** | | **B** |  |
| Daily | | Never | 0.180 | | 0.017 | | 10.480 | | 0.000 | | 0.146 | | 0.214 | | EFG |  |
| Daily | | Former | 0.119 | | 0.016 | | 7.490 | | 0.000 | | 0.088 | | 0.150 | | CDE |  |
| Daily | | Occasionally | 0.146 | | 0.022 | | 6.780 | | 0.000 | | 0.104 | | 0.188 | | CDEF |  |
| Daily | | Daily | 0.086 | | 0.023 | | 3.820 | | 0.000 | | 0.042 | | 0.131 | | ABCD |  |
| **Daily (mean)** | |  | **0.162** | | **0.015** | | **10.840** | | **0.000** | | **0.133** | | **0.192** | | **A** |  |

*Margins sharing a letter in the group label are not significantly different at the 5% level. Within and between dependent variables (Open to use e-cigarettes / Open to use sus). Mean values were tested separately. Bonferroni method
