# Supplementary material for: How do Smokers in a Snus-Prevalent Society Consider E-cigarettes, Snus, and Nicotine Replacement Therapy Products as Relevant Replacements for Cigarettes in the Event They Should Stop Smoking?
Source: Nicotine Tob Res. 2023 Jul 6;25(11):1753–61. doi: 10.1093/ntr/ntad113 (PMC10475606; doi:10.1093/ntr/ntad113)
Supplement: ntad113_suppl_Supplementary_Materials [file ntad113_suppl_supplementary_materials.zip › Supplementary file 1 - Figures S1 and S2.docx]

**Figure S1. Predicted probability of being open to use e-cigarettes or snus in the event of quitting smoking, by combinations of e-cigarette and snus use status**

**Figure S2. Predicted probability of being *not* open to use e-cigarettes or snus in the event of quitting smoking, by combinations of e-cigarette and snus use status**
