# Supplementary material for: How do Smokers in a Snus-Prevalent Society Consider E-cigarettes, Snus, and Nicotine Replacement Therapy Products as Relevant Replacements for Cigarettes in the Event They Should Stop Smoking?
Source: Nicotine Tob Res. 2023 Jul 6;25(11):1753–61. doi: 10.1093/ntr/ntad113 (PMC10475606; doi:10.1093/ntr/ntad113)
Supplement: ntad113_suppl_Supplementary_Materials [file ntad113_suppl_supplementary_materials.zip › Supplementary file 2 - Stata codes.docx]

*Stata codes for main model and figures

*Main model

gsem (ib3.open_ecigg_3 <- i.sex c.age i.education i.income i.number_household /// i.plans_to_quit i.smoking i.use_ecigg i.use_snus i.first_product_used) ///

(ib3.open_snus_3 <- i.sex c.age i.education i.income i.number_household /// i.plans_to_quit i.smoking i.use_ecigg i.use_snus i.first_product_used) ///

(ib3.open_nrt_3 <- i.sex c.age i.education i.income i.number_household /// i.plans_to_quit i.smoking i.use_ecigg i.use_snus i.first_product_used) ///

if smoking>=3, mlogit coeflegend vce(robust)

*Figure 1, all smokers:

margins,

predict(pr outcome(1.open_ecigg_3)) predict(pr outcome(2.open_ecigg_3)) ///

predict(pr outcome(3.open_ecigg_3)) ///

predict(pr outcome(1.open_snus_3)) predict(pr outcome(2.open_snus_3)) ///

predict(pr outcome(3.open_snus_3)) ///

predict(pr outcome(1.open_nrt_3)) predict(pr outcome(2.open_nrt_3)) ///

predict(pr outcome(3.open_nrt_3))

*Figure 1, smokers who had never used e-cigarettes or snus:

margins, at(use_ecigg=(4) use_snus=(4)) ///

predict(pr outcome(1.open_ecigg_3)) predict(pr outcome(2.open_ecigg_3)) ///

predict(pr outcome(3.open_ecigg_3)) ///

predict(pr outcome(1.open_snus_3)) predict(pr outcome(2.open_snus_3)) ///

predict(pr outcome(3.open_snus_3)) ///

predict(pr outcome(1.open_nrt_3)) predict(pr outcome(2.open_nrt_3)) ///

predict(pr outcome(3.open_nrt_3))

*Figure 1, daily and occasional smokers

margins smoking,

predict(pr outcome(1.open_ecigg_3)) predict(pr outcome(2.open_ecigg_3)) ///

predict(pr outcome(3.open_ecigg_3)) ///

predict(pr outcome(1.open_snus_3)) predict(pr outcome(2.open_snus_3)) ///

predict(pr outcome(3.open_snus_3)) ///

predict(pr outcome(1.open_nrt_3)) predict(pr outcome(2.open_nrt_3)) ///

predict(pr outcome(3.open_nrt_3))

*Figure 2

margins sex education income plans_to_quit first_product_used smoking use_snus use_ecigg, /// predict(equation(1.open_ecigg_3))

margins, at(age=(25(10)75)) predict(equation(1.open_ecigg_3))

margins sex education income plans_to_quit first_product_used smoking use_snus use_ecigg, /// predict(equation(1.open_snus_3))

margins, at(age=(25(10)75)) predict(equation(1.open_snus_3))

margins sex education income plans_to_quit first_product_used smoking use_snus use_ecigg, /// predict(equation(1.open_nrt_3))

margins, at(age=(25(10)75)) predict(equation(1.open_nrt_3))

***Figure S1 (Supplementary file 1)**

margins use_ecigg, at(use_snus=(1(1)4)) level(95) predict(pr outcome(1.open_snus_3))

margins use_snus, at(use_ecigg=(1(1)4)) level(95) predict(pr outcome(1.open_ecigg_3))

***Figure S2 (Supplementary file 1)**

margins use_ecigg, at(use_snus=(1(1)4)) level(95) predict(pr outcome(3.open_snus_3))

margins use_snus, at(use_ecigg=(1(1)4)) level(95) predict(pr outcome(3.open_ecigg_3))
